# Severe Periodontitis Is a Major Contributory Factor to Unmet Dental Healthcare Needs among Rheumatoid Arthritis Patients in Hong Kong

**DOI:** 10.1155/2022/8710880

**Published:** 2022-12-02

**Authors:** Mo Yin Mok, Wing Yin Kong, Katherine Chiu Man Leung, Mei Kuen Chow, Yi Lo, Claudia Pui Ling Tsang, Henry Kwok Kong Fung, Wallace Chak Sing Lau, Wai Keung Leung

**Affiliations:** ^1^Department of Biomedical Sciences, City University of Hong Kong, Kowloon Tong, Hong Kong; ^2^Faculty of Dentistry, University of Hong Kong, Pok Fu Lam, Hong Kong; ^3^School of Nursing, University of Hong Kong, Pok Fu Lam, Hong Kong; ^4^Department of Medicine, University of Hong Kong, Pok Fu Lam, Hong Kong; ^5^Department of Physiotherapy, Queen Mary Hospital, Pok Fu Lam, Hong Kong; ^6^Department of Occupational Therapy, Queen Mary Hospital, Pok Fu Lam, Hong Kong

## Abstract

**Objective:**

This study aimed to examine the frequency and predictive factors of adverse oral and dental outcomes in patients with rheumatoid arthritis (RA) with the goal to address their unmet dental healthcare needs in the metropolitan city of Hong Kong.

**Methods:**

238 RA patients followed up at local public hospitals were recruited in this cross-sectional study. A full dental examination was performed. Data were compared with the retrospective data collected from age-matched control groups in the community conducted in a territory-wide oral health survey in 2011. Predictive factors for severe periodontitis including various demographic and disease-specific factors were examined by multiple logistic regression analysis.

**Results:**

Loose teeth and gum bleeding were frequent dental complaints. Only 85.0% of RA patients had >20 natural teeth. Total edentulism was observed in 3.8% of patients, which was higher among adult (22–64 years) and elderly (>65 years old) RA patients than their respective age-matched community control groups. RA patients had a higher decayed, missing, and filled tooth score. Adult RA patients had a 5.3-fold increase in risk of severe periodontitis than their community counterparts. The plaque index was the main predisposing factor for severe periodontitis (odds ratio 17.5, *p*=0.001), which was worse among the 22–34 age group of patients. More RA patients required tooth extraction compared to dental filling for their community controls.

**Conclusion:**

Severe periodontitis is a major cause of unmet dental healthcare needs among RA patients in Hong Kong. It is recommended that dental care plans for RA patients be commenced early among newly diagnosed patients.

## 1. Introduction

The healthcare system of Hong Kong, as a globalised city in Asia, has achieved effective provision of health service, with 90% of the population served by the public health sector [1]. However, dental care in the territory is mainly provided by private dentists [2]. The World Health Organization (WHO) advocates the implementation of oral health programs in all countries [3], with the target to achieve retention of at least 20 natural teeth for proper dental function in older adults [4]. Although poor oral health is common among low-income countries [5], recent evidence has revealed an association between periodontitis and common chronic noncommunicable diseases such as diabetes mellitus [6] and cardiovascular disease [7] in developed countries.

Rheumatoid arthritis (RA) is a chronic inflammatory arthritic disease that shares similar predisposing factors and inflammatory mechanisms with chronic periodontitis [8]. An oral microbiome, which can be studied by high-throughput expression profiling nowadays [9], has been implicated as one of the triggering factors for RA [10]. Proinflammatory mediators found in the rheumatoid synovium such as those involved in NF-*κ*B and RANKL pathways have also been demonstrated in mucosal and periodontal inflammation [11, 12]. Severe periodontitis is one of the major causes of tooth loss and causes major public health issues and affects around 10–20% of the global population [13]. An increased risk of periodontitis has been shown in different cohorts of early and chronic RA patients compared to control groups comprising osteoarthritis patients [14, 15], non-RA dental and medical service recipients [16–18], and healthy subjects [19]. On the other hand, case-control comparison in some population-based databases showed no difference in risk [20, 21].

The aims of this study included examining dental and periodontal outcomes, dental health seeking behavior, and dental needs of RA patients in Hong Kong and examining disease-specific and nonspecific factors predictive for adverse dental and periodontal outcomes in these patients. Data examined in the recruited patients in this cross-sectional study were compared with the retrospective data collected from age-matched control groups in the general population reported in a territory-wide Oral Health Survey conducted by the Department of Health in Hong Kong [22], for which subjects in the community were sampled in accordance with index age groups categorised by the WHO [23]. Information generated from this study is essential in addressing unmet dental healthcare needs among RA patients so as to facilitate dental healthcare planning.

## 2. Methods

This study was a cross-sectional project approved by the Ethics Committee of the University of Hong Kong/Hospital Authority Hong Kong West Cluster (ethics approval number UW15-382).The inclusion criteria for subject recruitment included adult patients who satisfied the 2010 American College of Rheumatology/European League against Rheumatism classification criteria for RA [24]. Consecutive patients who consented to the study were recruited from the university-affiliated rheumatology clinic and rheumatology clinics at other public hospitals in Hong Kong from December 2016 to May 2018. The exclusion criteria included patients who declined to provide consent, who were institutionalised for mental diseases, and those who were fully dependent on their activities of daily living.

Demographic and clinical data of all patients were recorded. A standard examination for disease activity on 28 peripheral joints was performed by a rheumatologist (MYM) [25]. Patient (PatGH) and physician (PhyGH) global assessments were measured on a 10 cm visual analogue scale. The clinical disease activity index (CDAI) was evaluated by summation of the swollen joint count (SJC), tender joint count (TJC), PatGH, and PhyGH [26]. Grip strength was measured by using a sphygmomanometer on both hands. Schirmer's test was performed on both eyes and defined as positive for tear flow <5 mm in 5 minutes [27]. As a salivary gland biopsy was not routinely performed, secondary Sjogren's syndrome was diagnosed based on the modified European criteria proposed by the American/European Consensus Group [28], including dry eye and/or dry mouth symptoms for more than 3 months plus positive Schirmer's test and/or anti-Ro/La antibodies. A self-administered questionnaire was used to record dental complaints in the past 12 months and the use of dental prostheses.

### 2.1. Dental Examination and Dental Outcomes Examined

All patients except those who were totally edentulous underwent a full dental examination at the Prince Philip Dental Hospital. A dental examination was carried out by the same trained and calibrated dentist evaluator (KCML) in a standard dental surgery room using a mouth mirror and WHO probe. Oral hygiene was assessed using the plaque index (PI) [29], which was scored as follows: 0 = no plaque; 1 = tooth appears clean but plaque may be removed from its gingival third with a probe; 2 = moderate accumulation of plaque deposits visible to the naked eye; 3 = heavy accumulation of soft material filling the niche between the gingival margin and the tooth surface; it was calculated as the intraindividual mean from four sites per tooth. The number of teeth that were decayed (DT), missing (MT), or filled (FT), which made up the decayed, missing, filling (DMFT) score, was recorded according to the modified International Caries Detection and Assessment System [30]. Periodontal status was assessed using the Community Periodontal Index (CPI) using CPITN probes (WHO 621) on the index teeth. If the index teeth were missing on a sextant, all the remaining teeth in that sextant were considered. The deepest pocket depth category and the maximum loss of attachment (LOA) [31] were summated to obtain the CPI scores [32], which were defined as follows: 0 = healthy, 1 = bleeding after probing, 2 = calculus, 3 = periodontal pocket 4-5 mm, and 4 = periodontal pocket ≥6 mm. The deepest score among the 6 index teeth was selected. CPI >3 was classified as moderate periodontitis and CPI >4 as severe periodontitis.

### 2.2. Oral Health Survey

The Oral Health Survey was conducted by the Department of Health in Hong Kong in 2011 [22], six years apart from our current study. In accordance with the WHO categorisation, dental outcomes from five index age groups in the general population were sampled: 5-year-old children, 12-year-old students, 35–44-year-old adults, 65–74-year-old noninstitutionalised older persons, and elderly aged 74 and above [23]. Briefly, records of quarters in the frame of quarters maintained by the Census and Statistics Department were first sorted by the geographical area and type of quarters to achieve sample selection. 8,514 addresses of quarters were drawn systematically to form replicates according to a fixed sampling interval after selecting a random start number, and 17 replicates were selected. A total of 1,160 adults of 35–44-year-old living in these addresses were identified by household interviews, among whom 530 participated in the dental examination. The survey estimate was inferred to 1,062,900 persons in the general population in the government report. Similarly, from a randomly selected sample of 8,514 addresses, 1,108 noninstitutionalised older persons of age 65–74 years were identified and 576 participated in the dental examination. The estimate was inferred to 450,800 persons in the general population.

### 2.3. Categorisation of the Patient Cohort

Dental and periodontal data of 238 adult RA patients in this cohort were categorised in the same fashion as in the 2011 Oral Health Survey by 10-year age groups: 22–34, 35–44, 45–54, 55–64, 65–74, and >74. Dental data including DMFT and its components were collected from all RA patients, whereas gingival data collection excluded patients who were totally edentulous, for whom periodontal examination was not feasible. As sample sizes of patients belonging to 35–44 (6.3%, *n* = 15) and ≥65 (29.0%, *n* = 69) age groups were small, RA patients were further categorised into adult (aged 22–64 years) and elderly (≥65 year) groups for statistical comparison.

### 2.4. Statistical Analysis

Data analysis was performed using SPSS 27.0 (California, USA). Data were expressed as a mean ± standard deviation (SD) unless otherwise specified. The chi-square test and Fisher's exact test where appropriate were performed for comparison of categorical data between groups. Bonferroni's correction was applied for multiple comparison. Multiple logistic regression analysis was performed using severe periodontitis as the dependent variable and variables identified in univariate analysis with *p* < 0.1 as independent factors, using an enter regression model. Odds ratios (ORs) with 95% confidence intervals (CIs) were determined. A *P* value less than 0.05 was considered statistically significant.

## 3. Results

### 3.1. Demographic and Clinical Characteristics of Patients

**Figure 1:**
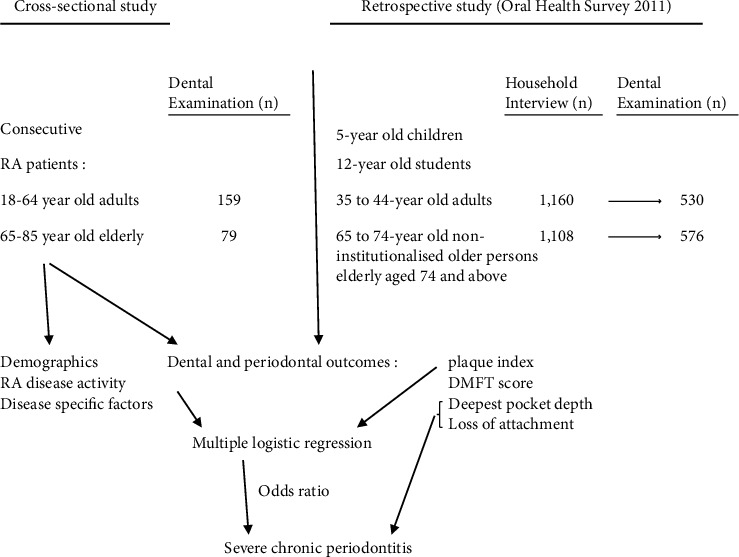
Diagram representation of the study design and outcomes.

Two hundred and thirty-eight (223 female and 15 male) RA patients of southern Chinese ethnicity were recruited.  Figure 1 shows a diagram representation of the study design and outcomes. Raw data presented in Tables S1–S4 and Figure S1 can be found in the Supplementary file.  Table 1 shows demographic and clinical characteristics of patients. The age of these patients was 58.8 ± 10.8 years with disease duration of 15.1 ± 11.0 years. Patients aged 22–64 years and ≥65 years were categorised into adult (66.8%, *n* = 159) and elderly (33.2%, *n* = 79) groups, respectively. Smoking history was recorded in 5.5% of patients compared to 12-13% of community subjects reported in the Oral Health Survey. The serum rheumatoid factor and anti-CCP antibodies were found in 74.8% and 67.7% of patients, respectively. Secondary Sjogren's syndrome was diagnosed in 26.9% of patients. The majority of patients had active RA, among whom 28.2% had moderate-to-high disease activity (CDAI >10).

### 3.2. Dental Complaints

Dental symptoms were frequent and included tooth hypersensitivity (61.2%), gum bleeding (59.0%), and loose teeth (43.7%) (Table 2). Compared with their community counterparts, RA patients in the adult and elderly groups had more complaints of loose teeth and gum bleeding, respectively.

### 3.3. Oral Condition

Clinical xerostomia was found in 19.9% of patients, including stickiness of the mouth mirror to cheek mucosa (12.5%), dryness (18.5%), and atrophic tongue (7.1%). Other mucosal features such as angular cheilitis (1.2%), oral candidiasis (1.2%), and white patches (3.6%) were also observed. The PI of these patients was 0.73 ± 0.19. The 22–34 age group had the worst PI (0.80 ± 0.15).

### 3.4. Dental Disease

Only 85.0% of RA patients had more than 20 standing teeth (24.8 ± 7.1, range 0–32). Adult and elderly RA patients had 26.7 ± 5.5 and 21.0 ± 8.4 teeth compared to 28.6 and 19.3 teeth in the 35–44 and 65–74 age groups in the community, respectively. The proportion of adult RA patients who had more than 20 teeth was lower than that of their community counterparts (93.7% vs 99.8%). Furthermore, 9 patients (3.8%) were totally edentulous involving 2.5% (*n* = 4) and 6.3% (*n* = 5) of the adult and elderly groups compared with 0% and 5.6% of their community counterparts, respectively. Disease duration of edentulous RA patients was 15.0 ± 6.9 years. The youngest edentulous patient was a 55-year-old woman with disease duration of 22 years.

**Figure 2:**
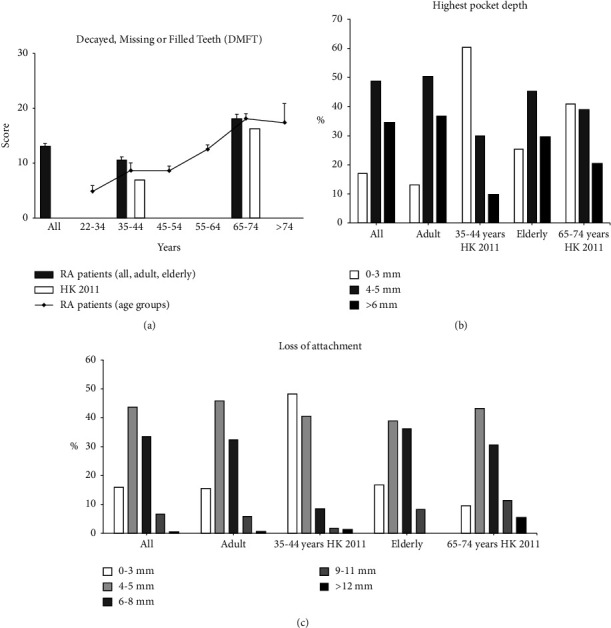
Dental and periodontal outcomes of rheumatoid arthritis patients. (a) DMFT score of RA patients by 10-year age groups (line representation) and by adult and elderly categories in comparison with age-matched community subjects in the 2011 Oral Health Survey (bar representation). (b) Percentage of RA patients with different extent of the highest pocket depth and (c) loss of attachment by adult and elderly categories.

Table 2 summarises the dental outcomes of RA patients and their community counterparts reported in the Oral Health Survey. The DMFT score of RA patients was 12.8 ± 8.1, affecting 97.9% of patients. Adult RA patients were found to have higher DMFT scores (10.3 ± 6.9 vs 6.9), while elderly RA patients had slightly higher DMFT (18.0 ± 7.8 vs 16.2) than their community controls. A direct comparison of RA patients belonging to 35–44 (*n* = 15) and 65–74 (*n* = 69) age groups with their respective controls also showed that RA patients had higher DMFT scores. The DMFT scores of RA patients and control subjects are shown in Figure 2(a).

Adult RA patients were found to have a higher ratio of MT to DT scores than their community counterparts (5.4/0.5 vs 3.4/0.7). There was a higher proportion of adult RA patients having MT (93.1% vs 89.7%), while less having DT (25.2% vs 31.2%). A direct comparison of RA patients belonging to the 35–44 age group (*n* = 15) with their age-matched controls also showed this pattern of higher MT but lower DT scores (3.5/0.9 vs 3.4/0.7). MT and DT scores were not particularly different between elderly RA patients and their controls. A remarkably higher number of filled teeth (FT) with a higher proportion of affected subjects were observed in both adult and elderly RA patients compared to the general population. A direct comparison of RA patients belonging to the 35–44 (4.3 in 80.0% vs 2.8 in 67.4%) and ≥65 (6.2 in 84.1% vs 2.3 for 59.5%) age groups with their community counterparts also showed this pattern of higher FT with a higher proportion of subjects affected.

### 3.5. Gingival and Periodontal Disease

Table 2 summarises the periodontal outcomes of RA patients and their community counterparts. A higher proportion of adult RA patients had a deepest pocket depth of ≥4 mm (88.2% vs 39.6%) and ≥6 mm (36.6% vs 9.8%) than their community controls (Figure 2(b)). A higher proportion of elderly RA patients also had a deepest pocket depth of ≥4 mm (76.1% vs 59.1%) than their controls, but the proportion of subjects having a deepest pocket depth of ≥6 mm was not particularly different. A direct comparison of RA patients belonging to the 35–44 (*n* = 15) and 65–74 (*n* = 63) age groups with their community counterparts also showed the same pattern. Similarly, significantly more adult RA patients had LOA by ≥4 mm (85.2% vs 51.8%, *p* < 0.001), ≥6 mm (38.7% vs 11.3%, *p* < 0.001), and ≥9 mm (7.7% vs 2.9%, *p*=0.007) than their community counterparts, but no difference in LOA was observed between elderly RA patients and their controls (Figure 2(c)).

Adult RA patients were found to have a 11-fold increase in risk of moderate periodontitis (CPI >3, deepest gum pocket ≥4 mm) (OR 11.43 (95% CI 6.78–19.26), *p* < 0.001) and a 5-fold increase in risk of severe periodontitis (defined by CPI >4, deepest gum pocket ≥6 mm) (OR 5.31 (95% CI 3.43–8.21), *p* < 0.001) compared to their community counterparts. Higher risk of moderate periodontitis was also found in elderly RA patients than in community elderly (OR 2.20 [95% CI 1.23–3.89], *p*=0.006), but no difference in frequency of severe periodontitis was observed.

### 3.6. Association of Dental and Gingival Conditions with Disease-Specific Factors

Scores of DMFT and individual components were found to correlate with age (*r* = 0.459, *p* < 0.001). The PI was shown to correlate with the deepest pocket depth (*r* = 0.427, *p* < 0.001) and LOA (*r* = 0.274, *p* < 0.001) and was associated with severe periodontitis in RA patients (0.79 ± 0.14 vs 0.70 ± 0.21, *p* < 0.001). Severe periodontitis was also shown to be associated with the higher CDAI (58.09 ± 35.97 vs 46.86 ± 31.92, *p*=0.02). Logistic regression analysis revealed that the PI contributed to a 17-fold increase in risk of severe periodontitis in RA patients (OR 17.48 (95% CI 3.06–99.74), *p*=0.001), whereas CDAI contributed only to a modest increase in risk (OR 1.01 (95% CI 1.002–1.019), *p*=0.02). Dental and gingival outcomes were not found to be associated with other disease-specific factors, including disease duration, autoantibody profile, secondary Sjogren's syndrome, and parameters of disease activity after Bonferroni's correction.

### 3.7. Assessed Dental Treatment Needs

Twenty-five patients reported regular use of dental prostheses (10.5%) including removable partial dentures (*n* = 24) and dental bridge (*n* = 1). Oral hygiene instruction was prescribed by attending dentists to all RA patients (Table 3). More adult RA patients were recommended for dental extraction (20.4% vs 11.9%), whereas most of their community counterparts were advised for dental filling or root canal treatment (29.0% vs 18.4%).

### 3.8. Utilisation of Dental Healthcare Services

In terms of utilisation habits of dental healthcare services, dental visits were more frequent in community subjects than in RA patients (every 6 months vs every 1-2 years). There remained 5.5% of RA patients who had never visited a dentist.

## 4. Discussion

Our study revealed that dental healthcare issues of RA patients are an unmet need in Hong Kong and dominated by severe periodontal disease. Retention of at least 20 natural teeth for proper dental function for older adults is the overarching principle behind all worldwide oral health programs advocated by the WHO [3]. We found that only 85.0% of RA patients in our cohort with a mean age of 58.8 years had 20 teeth or more. Total edentulism was recorded in 3.8% of patients, which was higher among adult and elderly patients than their community counterparts. Indeed, RA patients were reported to have a 2-fold increase in risk of edentulism in previous studies [33]. Tooth loss in RA patients occurred at younger age [21] and early in the disease course [34]. This is likely related to worse dental and periodontal outcomes as were observed in our RA patients compared with age-matched community subjects, and the differences of which were already apparent in younger patients of the 35–44 age group.

Our RA patients had a higher DMFT score than the general population compatible with worse dental condition. Adult RA patients had more missing but less decayed teeth than age-matched community subjects. Both adult and elderly RA patients also had remarkably more filled teeth, suggestive of worse dental decay issues. Missing teeth were a more dominant tooth problem in RA patients reported in the literature than their adverse dental decay experience [33]. Missing teeth may be a result of dental extraction as a treatment to decayed teeth or loose teeth from severe periodontitis. Indeed, loose teeth was a frequent dental complaint of our patients and needs assessment of our cohort revealed that more RA patients were offered advice on dental extraction compared to more advice on root canal treatment and dental filling given to community subjects.

Our adult and elderly RA patients were found to have an increased risk of moderate periodontitis by 11-fold and 2-fold compared to their community counterparts, respectively. Severe periodontitis was found in 36.6% of adult RA patients who had a 5-fold increase in risk compared to age-matched community subjects. The absence of difference in frequency of severe periodontitis between elderly RA patients and controls may be due to the higher rate of total edentulism in RA patients, masking the presence of periodontitis as the underlying cause. Increased frequency of periodontitis in RA patients has been reported in case-controlled studies in Western [14–16, 18, 35] and Asian countries [17, 19, 36, 37], which ranged from 27% to 63.6%, depending on the criteria applied to define periodontitis except for a negative finding reported in a few studies [21, 37].

Cigarette smoking is a shared predisposing factor for both RA and periodontitis [38]. Smoking has been demonstrated to be a strong risk factor for periodontal disease in RA patients [20], particularly those who were seropositive for anti-CCP antibodies [39]. Unlike other Asian cohorts, which comprised more smokers [36], we did not find any association between periodontitis and cigarette smoking. Secondary Sjogren's syndrome reported in 26.9% of our patients was not found to correlate with any dental outcomes after Bonferroni's correction in statistical analysis [40].

We found that the PI was the main predictive factor for severe periodontitis in our RA patients, contributing to an increase in risk by 17-fold. It is noteworthy that the 22–34 age group in our RA cohort had the worst PI score. Another study also reported an association between dental plaque and periodontitis in RA patients [36]. The gingival biotype also impacts the clinical response of dental interventions [41]. On the other hand, other studies revealed that RA disease was another independent risk factor for periodontitis [42] after correction with the oral hygiene status in statistical analysis [16]. Deeper periodontal pockets have been reported in patients with early RA who had comparable oral hygiene status as the control group [34].

There has been increasing evidence that suggests a bidirectional relationship between RA and periodontitis [42]. Mucosal inflammation associated with altered microbial diversity and profile in the oral cavity and gastrointestinal and respiratory tracts have been implicated in RA pathogenesis [10]. The subgingival microbiome examined by high-throughput expression profiling techniques, such as metagenomics and 16S gene analysis, has been demonstrated to differ between RA patients and non-RA subjects [43] and smoking status [9]. Proinflammatory mediators expressed in the rheumatoid synovium such as IL-6, TNF-*α*, transglutaminase 2, and inflammasome Nlrp3 [12] have also been detected in saliva and serve as biomarkers for periodontal disease [11].

Disease-specific factors associated with chronic RA such as temporomandibular [44] and upper limb joint involvement [45] with impaired hand grip strength may contribute to inefficient toothbrushing [46], although we did not find any correlation between dental outcomes and these clinical features. Inconsistent findings have been reported in the association between various dental outcomes and disease-specific parameters in different studies, which likely reflected the varied composition of these study cohorts. Severe periodontitis was shown to be associated with serum anti-CCP antibodies and rheumatoid factor in some studies [14, 15, 47] but not others [16, 18, 20]. Higher DAS28 was reported to correlate with DMFT [48] and periodontitis in some studies [18, 47] but was not consistently demonstrated [15]. Although we found an association between the CDAI and severe periodontitis, the risk contributed by the CDAI was only 1.01-fold, suggesting that the contributory effect of systemic inflammation was only modest compared to local gingival inflammation associated with dental plaque.

We identified a lack of patient awareness and suboptimal dental health seeking behavior as social factors that also accounted for poor dental health in our RA patients. Unlike community subjects, RA patients visited dentists rarely, and there remained 5.5% of patients who had never visited a dentist. This finding was echoed by a National Health Survey in Australia, which demonstrated that RA patients were less likely to have visited a dental professional [49]. Thus, modification of health seeking behavior is a crucial strategic plan to improve oral health outcomes in RA patients.

Our study was limited by the lack of a concomitant control group but relied on comparison with retrospective data obtained from a territory-wide community survey performed 6 years apart. Despite the restricted study design, we demonstrated worse dental outcomes in our RA patients with an increased risk of moderate and severe periodontitis in accordance with the findings of other studies [50].

As we revealed that RA patients are at risk of severe periodontitis with the manifestation of adverse risk factors and dental outcomes at a relatively young age compared to their community counterparts, healthcare authorities may provide patient education preferably at the time of diagnosis of RA or early in the disease course so as to raise patient awareness on dental health and to encourage regular dental health seeking behavior. A multidisciplinary approach can empower RA patients with better self-care. This may involve counseling on cigarette cessation by nurses, education on proper use of dental cleaning devices, adoption of compensatory behavior to achieve efficient toothbrushing [51], preparation of splints or assistive adaptors by physiotherapists, and upper limb power training by occupational therapists. Regular scaling can be offered by dental healthcare workers so as to eliminate barriers of dental seeking behavior [52]. For patients with established RA in whom we reported a prominent issue of tooth loss, there remains an unmet need for the provision of secondary dental care services in the public sector in Hong Kong. Patient education can be provided by the government in regard to the choices of dentures and dental prostheses, techniques for proper application, and denture hygiene.

In conclusion, we showed that tooth loss and severe periodontitis were more frequent among RA patients than their community counterparts in Hong Kong, contributing to major unmet needs in our dental healthcare. Unfavorable dental risk factors and outcomes were observed among RA patients at a relatively young age. A poor plaque index was the main factor that predisposed to periodontitis in these patients. Dental healthcare providers may consider care pathways that commence early for newly diagnosed patients with emphasis on preventive measures in addition to provision of dental intervention and prosthesis for patients with established RA. Optimisation of care plans tailored to unmet needs in chronic disease groups can tackle the growing dental healthcare burden and attain the goal of keeping good dental function and nutrition as advocated by the WHO.

## Figures and Tables

**Table 1:** Demographic and clinical characteristics of rheumatoid arthritis patients in this study (*n* = 238).

Demographic and clinical characteristics	Number (percentage)	Mean ± standard deviation; IQR
Gender (female)	223/238 (93.7%)	
Age (years)		58.8 ± 10.8 (range 22–85)
Subcategories		
Adults (age 22–64 years)	159/238 (66.8%)	
Elderly (age ≥65 years)	79/238 (33.2%)	
Duration of the disease (years)		15.1 ± 11.0 (range 1–55)
Current smoker or ex-smoker	13/238 (5.5%)	
Autoantibodies		
Rheumatoid factor	178/238 (74.8%)	
Anti-CCP antibodies	128/189 (67.7%)	
Anti-Ro antibodies	19/104 (18.3%)	
Probable secondary Sjogren's syndrome	64/238 (26.9%)	
Dry eyes and mouth >3 months	140/238 (58.8%)	
Positive Schirmer's test (<5 mm in 5 minutes)	50/238 (21.0%)	
Anti-Ro/La antibodies	43/195 (22.1%)	
Disease activity		
Swollen joint count (SJC)		1.4 ± 2.2 (median 1, range 0–20)
Tender joint count (TJC)		1.5 ± 2.5 (median 0, range 0–14)
Patient general health (PatGH)		3.8 ± 2.4 (median 4.0, range 0–10)
Physician global health (PhyGH)		1.1 ± 1.5 (median 0.7, range 0–8.4)
Clinical disease activity index (CDAI)		7.8 ± 6.2 (median 6.5, range 0–40.4)
Active disease (CDAI >2.8)	197/238 (82.8%)	
Moderate and high disease activity (CDAI >10)	67/238 (28.2%)	
Temporomandibular joint pain and stiffness	25/238 (10.5%)	
Grip strength (mmHg)		
Left hand	62.5 ± 41.6	
Right hand	63.9 ± 43.0	
Treatment		
Disease modifying antirheumatic drugs	212/238 (89.1%)	
Methotrexate	151/238 (63.4%)	
Hydroxychloroquine	94/238 (39.7%)	
Sulfasalazine	62/238 (26.1%)	
Leflunomide	30/238 (12.6%)	
Oral prednisolone	26/238 (10.9%)	
Biologic agents	32/238 (13.4%)	

IQR, interquartile range.

**Table 2:** Summary of dental and periodontal outcomes in rheumatoid arthritis patients and age-matched community subjects reported in the 2011 Oral Health Survey.

Oral and dental outcomes	RA patients	RA patients	Oral health survey 2011	OR (95% CI), *p*value	RA patients	Oral Health Survey 2011, noninstitutionalised, 65–74 year olds	OR (95% CI), *p*value
	All	Adult group	35–44 year olds		Elderly group		
Dental symptoms							
Sample size (*n*)	183	113	1,160		70	1,108	
Gum bleeding (%)	59.0	63.7	60.3		51.4	32.8	
Tooth hypersensitivity (%)	61.2	68.1	55.7		50.0	47.6	
Loose teeth (%)	43.7	44.2	14.8		42.9	41.9	
Toothache (%)	10.9	8.8	9.7		14.3	14.0	
Halitosis (%)	9.3	9.7	75.8		8.6	51.1	
Dental cleanliness							
PI score	0.73 ± 0.19	0.73 ± 0.18			0.72 ± 0.22		
Dental disease							
Sample size	238	159			79		
Total edentulism (%)	3.8	2.5	0		6.3	5.6	
Number of teeth (*n*)	24.8 ± 7.1	26.7 ± 5.5	28.6		21.0 ± 8.4	19.3	
Tooth >20 (%)	85.0	93.7	99.8		70.9	59.5	
DMFT score	12.8 ± 8.1	10.3 ± 6.9	6.9		18.0 ± 7.8	16.2	
DMFT (%)	97.9	96.9	96.1		100	99.3	
DT score	0.8 ± 1.5	0.5 ± 1.0	0.7		1.4 ± 2.1	1.3	
DT (%)	34.0	25.2	31.2		51.9	47.8	
MT score	7.2 ± 7.1	5.4 ± 5.5	3.4		11.0 ± 8.4	12.7	
MT (%)	95.0	93.1	89.7		98.7	98.1	
FT score	4.9 ± 4.4	4.4 ± 4.0	2.8		5.8 ± 5.0	2.3	
FT (%)	83.6	84.3	67.4		82.3	59.5	
Gingival and periodontal disease							
Sample size	224	153	530		71	576	
Highest pocket depth (%)							
>4 mm	84.3	88.2	39.6	11.43 (6.78–19.26), <0.001	76.1	59.1 20.4	2.20 (1.23–3.89), 0.006
≥6 mm	33.9	36.6	9.8	5.31 (3.43–8.21), <0.001	28.2		1.53 (0.88–2.67), 0.13
Loss of attachment (%)							
≥4 mm	84.6	85.2	51.8	5.34 (3.32–8.58), <0.001	83.3	90.5	0.53 (0.27–1.04), 0.06
≥6 mm	40.1	38.7	11.3	4.95 (3.24–7.54), <0.001	43.1	47.2	0.85 (0.52–1.39), 0.50
≥9 mm	7.9	7.7	2.9	2.83 (1.30–6.18), 0.007	8.3	16.7	0.46 (0.19–1.08), 0.07
≥12 mm	0.4	0.6	1.2	0.56 (0.07–4.67), 1.00	0	5.4	0.04

**Table 3:** Assessed dental treatment needs in rheumatoid arthritis patients and age-matched community subjects.

Dental treatment needed	All RA patients	Adult RA patients, *n* = 103	Elderly RA patients, *n* = 65	Oral Health Survey 2011
	Number (percentage)			
Scaling	146/168 (86.9%)	93 (90.3%)	53 (81.5%)	95.9%^1^/95.5%^2^
Root canal treatment/filling	47/168 (28.0%)	19 (18.4%)	28 (43.1%)	29.0%^1^/39.8%^2^
Extractions	37/168 (22.0%)	21 (20.4%)	16 (24.6%)	11.9%^1^/28.2%^2^
Tooth replacement by prosthesis	21/168 (12.5%)	9 (8.7%)	12 (18.5%)	8.2%^1^/25.4%^2^

^1^Age group 35–44; ^2^age group 65–74.

## Data Availability

The raw data used to support the findings of this study are provided in the supplementary file.

## Supplementary Materials

Supplementary Materials Supplementary Table S1: number of teeth in rheumatoid arthritis patients and age-matched community subjects. Supplementary Table S2A: dental outcomes of rheumatoid arthritis patients and age-matched community subjects by adult and elderly categories. Supplementary Table S2B: dental outcomes of rheumatoid arthritis patients and age-matched community subjects by 10-year age groups. Supplementary Table S3A: periodontal outcomes of rheumatoid arthritis patients and age-matched community subjects by adult and elderly categories. Supplementary Table S3B: periodontal outcomes of rheumatoid arthritis patients and age-matched community subjects by 10-year age groups. Supplementary Table S4: utilisation of dental healthcare services by rheumatoid arthritis and community subjects. Supplementary Figure S1: proportion of rheumatoid arthritis patients having positive DMFT (A), decayed teeth (B), missing teeth (C), and filled teeth (D) by age groups (line representation) and by adult and elderly categories (bar representation), in comparison with age-matched community subjects in the 2011 Oral Health Survey. Supplementary Figure S2: highest pocket depth (A) and maximum loss of attachment (B) in rheumatoid arthritis patients by 10-year age groups in comparison with age-matched community subjects in the 2011 Oral Health Survey.
