# Supplementary material for: Severe Periodontitis Is a Major Contributory Factor to Unmet Dental Healthcare Needs among Rheumatoid Arthritis Patients in Hong Kong
Source: Int J Dent. 2022 Dec 2;2022:8710880. doi: 10.1155/2022/8710880 (PMC9733996; doi:10.1155/2022/8710880)

Supplementary Table S1. Number of teeth in rheumatoid arthritis patients and age-matched community subjects.

Age subgroups	Number	RA patients			Oral Health Survey 2011		
		Number of teeth	Number (Percentage) >20 teeth	Total edentulous	Number of teeth	Percentage >20 teeth	Total edentulous
All	238	24.8 ± 7.1 (range 0-32)	200 (85.0%)	9 (3.8%)			
22-34 years	9 (3.8%)	30.2 ± 1.5 (range 28-32)	9 (100%)	0 (0%)			
35-44 years	15 (6.3%)	28.5 ± 3.0 (range 22-32)	15 (100%)	0 (0%)	28.6	99.8%	0%
45-54 years	47 (19.7%)	28.2 ± 2.4 (range 21-32)	47 (100%)	0 (0%)			
55-64 years	88 (37.0%)	25.1 ± 6.7 (range 0-32)	78 (88.6%)	4 (4.5%)			
65-74 years	69 (29.0%)	21.4 ± 7.9 (range 0-31)	50 (72.5%)	3 (4.3%)	19.3	59.5%	5.6%
>74 years	10 (4.2%)	18.3 ± 11.8 (range 0-32)	6 (60.0%)	2 (20.0%)			
Adult	159 (66.8%)	26.7 ± 5.5 (range 0-32)	149 (93.7%)	4 (2.5%)			
Elderly	79 (33.2%)	21.0 ± 8.4 (range 0-32)	56 (70.9%)	5 (6.3%)			

Supplementary Table S2A. Dental outcomes of rheumatoid arthritis patients (n=238) and age-matched community subjects by adult and elderly categories.

**Dental decay**	**This project**	**Oral Health Survey 2011**
	**Number/Percentage of patients (Score)**	**Percentage of subjects (Score)**
**Decayed, Missing or Filled Teeth (DMFT)**		
All	235/238 (97.9%) [score 12.8 + 8.1 (median 11, IQR 10, range 0-32)]	-
Adult	154/159 (96.9%) [score 10.3 + 6.9 (median 9, IQR 8, range 0-32]	96.1% [score 6.9]
Elderly	79/79 (100%) [score 18.0 + 7.8 (median 18, IQR 13, range 1-32]	99.3% [score 16.2]
**Decayed teeth (DT)**		
All	81/238 (34.0%) [score 0.8 + 1.5 (median 0, IQR 1, range 0-12)]	-
Adult	40/159 (25.2%) [score 0.5 + 1.0 (median 0, IQR 1, range 0-5)]	31.2% [score 0.7]
Elderly	41/79 (51.9%) [score 1.4 + 2.1 (median 1, IQR 2, range 0-12]]	47.8% [score 1.3]
**Missing teeth (MT)**		
All	226/238 (95.0%) [score 7.2 + 7.1 (median 5, IQR 5, range 0-32)]	-
Adult	148/159 (93.1%) [score 5.4 + 5.5 (median 4, IQR 4, range 0-32)]	89.7% [score 3.4]
Elderly	78/79 (98.7%) [score 11.0 + 8.4 (median 9, IQR 9, range 0-32]]	98.1% [score 12.7]
**Filled teeth (FT)**		
All	199/238 (83.6%) [score 4.9 + 4.4 (median 4, IQR 6, range 0-22)]	-
Adult	134/159 (84.3%) [score 4.4 + 4.0 (median 4, IQR 6, range 0-20)]	67.4% [score 2.8]
Elderly	65/79 (82.3%) [score 5.8 + 5.0 (median 6, IQR 6, range 0-22)]	59.5% [score 2.3]
**Edentulous**		
All	9/238 (3.8%)	-
Adult	4/159 (2.5%)	0%
Elderly	5/79 (6.3%)	5.6%

Supplementary Table S2B. Dental outcomes of rheumatoid arthritis patients (n=238) and age-matched community subjects by 10-year age groups.

Dental decay	This project	Oral Health Survey 2011
	**Number/Percentage of patients (Score)**	**Percentage of subjects (Score)**
**Decayed, Missing or Filled Teeth (DMFT)**		
All	235/238 (97.9%)<br>[score 13.0 ± 8.2 (median 11.0, IQR 11, range 0-32)]	-
22-34 years	8/9 (88.9%)<br>[score 4.9 ± 3.0 (median 5, IQR 5, range 0-9)]	-
35-44 years	14/15 (93.3%)<br>[score 8.7 ± 5.0 (median 9.0, IQR 7, range 0-18)]	96.1%<br>[score 6.9]
45-54 years	44/47 (93.6%)<br>[score 8.1 ± 5.1 (median 7, IQR 8, range 0-32)]	-
55-64 years	88/88 (100%)<br>[score 12.3 ± 7.6, median 11, IQR 8, range 1-32]	-
65-74 years	69/69 (100%)<br>[score 18.1 ± 7.4 (median 17, IQR 13, range 1-32)]	99.3%<br>[score 16.2]
>74 years	10/10 (100%)<br>[score 17.4 ± 10.8 (median 18, IQR 18, range 1-32)]	-
**Decayed teeth (DT)**		
All	81/238 (34.0%)<br>[score 0.8 ± 1.5 (median 0, IQR 1, range 0-12)]	-
22-34 years	2/9 (22.2%)<br>[score 0.6 ± 1.1 (median 0, IQR 1, range 0-3)]	-
35-44 years	6/15 (40.0%)<br>[score 0.9 ± 1.2 (median 0, IQR 2, range 0-4)]	31.2%<br>[score 0.7]
45-54 years	12/47 (25.5%)<br>[score 0.4 ± 0.9 (median 0, IQR 1, range 0-5)]	-
55-64 years	20/88 (22.7%)<br>[score 0.5 ± 1.1, median 0, IQR 0, range 0-4]	-
65-74 years	37/69 (53.6%)<br>[score 1.6 ± 2.2 (median 1, IQR 3, range 0-12)]	47.8%<br>[score 1.3]
>74 years	4/10 (40.0%)<br>[score 0.5 ± 0.7 (median 0, IQR 1, range 0-2)]	-
**Missing teeth (MT)**		
All	226/238 (95.0%)<br>[score7.4 ± 7.4 (median 5, IQR 6, range 0-32)]	-
22-34 years	7/9 (77.8%)<br>[score 1.8 ± 1.5 (median 1, IQR 3, range 0-4)]	-
35-44 years	12/15 (80.0%)<br>[score 3.5 ± 3.0 (median 3.0, IQR 4, range 0-10)]	89.7%<br>[score 3.4]
45-54 years	43/47 (91.5%)<br>[score 3.8 ± 2.4 (median 4, IQR 3, range 0-11)]	-
55-64 years	86/98 (97.8%)<br>[score 6.9 ± 6.7 (median 5, IQR 3, range 0-32)]	-
65-74 years	69/69 (100%)<br>[score 10.6 ± 8.0 (median 8, IQR 9, range 1-32)]	98.1%<br>[score 12.7]

>74 years	9/10 (90.0%)<br>[score 13.7 + 11.8 (median 10.5, IQR 22, range 0-32)]	-
**Filled teeth (FT)**		
All	199/238 (83.6%)<br>[score 4.8 + 4.4 (median 4, IQR 6, range 0-22)]	-
22-34 years	8/9 (88.9%)<br>[score 2.6 + 1.7 (median 2, IQR 3, range 0-5)]	-
35-44 years	12/15 (80.0%)<br>[score 4.3 + 3.6 (median 4, IQR 5, range 0-12)]	67.4%<br>[score 2.8]
45-54 years	36/47 (76.6%)<br>[score 3.9 + 3.6 (median 4, IQR 6, range 0-13)]	-
55-64 years	78/88 (88.6%)<br>[score 4.9 + 4.4 (median 4, IQR 6, range 0-20)]	-
65-74 years	58/69 (84.1%)<br>[score 6.2 + 5.1 (median 6, IQR 7, range 0-22)]	59.5%<br>[score 2.3]
>74 years	7/10 (70.0%)<br>[score 3.2 + 3.2 (median 2, IQR 7, range 0-7)]	-
**Edentulous**		
All	9/238 (3.8%)	-
22-34 years	0/9 (0%)	-
35-44 years	0/15 (0%)	0%
45-54 years	0/47 (0%)	-
55-64 years	4/88 (4.5%)	-
65-74 years	3/69 (4.3%)	5.6%
>74 years	2/10 (20.0%)	-

Supplementary Table S3A. Periodontal outcomes of rheumatoid arthritis patients (n=238) and age-matched community subjects by adult and elderly categories.

Gum Disease	Number (Percentage)	
	RA patients	Oral Health Survey 2011
**Highest pocket depth**		
0-3mm		
All	35/224 (15.6%)	-
Adult	18/153 (11.8%)	60.4%
Elderly	17/71 (23.9%)	40.8%
4-5mm		
All	113/224 (50.4%)	-
Adult	79/153 (51.6%)	29.8%
Elderly	34/71 (47.9%)	38.8%
≥6mm		
All	76/224 (33.9%)	-
Adult	56/153 (36.6%)	9.8%
Elderly	20/71 (28.2%)	20.4%
**Loss of attachment (LOA)**		
0-3mm		
All	35/227 (15.4%)	-
Adult	23/155 (14.8%)	48.2%
Elderly	12/72 (16.7%)	9.5%
4-5mm		
All	101/227 (44.5%)	-
Adult	72/155 (46.5%)	40.5%
Elderly	29/72 (40.3%)	43.2%
6-8mm		
All	73/227 (32.2%)	-
Adult	48/155 (31.0%)	8.4%
Elderly	25/72 (34.7%)	30.6%
9-11mm		
All	17/227 (7.5%)	-
Adult	11/155 (7.1%)	1.7%
Elderly	6/72 (8.3%)	11.4%
≥12mm		
All	1/227 (0.4%)	-
Adult	1/155 (0.6%)	1.2%
Elderly	0/72 (0%)	5.4%
**Chronic periodontitis** (CPI ≥4)		
**All**	90/227 (39.6%)	
Adult	64/155 (41.3%)	
Elderly	26/72 (36.1%)	

Supplementary Table S3B. Periodontal outcomes of rheumatoid arthritis patients and age-matched community subjects by 10-year age groups.

Gum Disease	Number (Percentage)	
	RA patients	Oral Health Survey 2011
**Highest pocket depth**		
0-3mm		
All	38/224 (17.0%)	-
22-34 years	1/9 (11.1%)	-
35-44 years	1/15 (6.7%)	60.4%
45-54 years	5/47 (10.6%)	-
55-64 years	13/82 (15.9%)	-
65-74 years	16/63 (25.4%)	40.8%
>74 years	2/8 (25.0%)	-
4-5mm		
All	109/224 (48.7%)	-
22-34 years	6/9 (66.7%)	-
35-44 years	8/15 (53.3%)	29.8%
45-54 years	24/47 (51.1%)	-
55-64 years	39/82 (47.6%)	-
65-74 years	28/63 (44.4%)	38.8%
>74 years	4/8 (50.0%)	-
≥6mm		
All	77/224 (34.4%)	-
22-34 years	2/9 (22.2%)	-
35-44 years	6/15 (40.0%)	9.8%
45-54 years	18/47 (38.3%)	-
55-64 years	30/82 (36.6%)	-
65-74 years	19/63 (30.2%)	20.4%
>74 years	2/8 (25.0%)	-
**Loss of attachment**		
0-3mm		
All	36/227 (15.9%)	-
22-34 years	1/9 (11.1%)	-
35-44 years	1/15 (6.7%)	48.2%
45-54 years	10/47 (21.3%)	-
55-64 years	12/84 (14.3%)	-
65-74 years	11/64 (17.2%)	9.5%
>74 years	1/8 (12.5%)	-
4-5mm		
All	99/227 (43.6%)	-
22-34 years	6/9 (66.7%)	-
35-44 years	8/15 (53.3%)	40.5%
45-54 years	20/47 (42.6%)	-
55-64 years	37/84 (44.0%)	-
65-74 years	25/64 (39.1%)	43.2%
>74 years	3/8 (37.5%)	-
6-8mm		
All	76/227 (33.5%)	-

22-34 years	2/9 (22.2%)	-
35-44 years	6/15 (40.0%)	8.4%
45-54 years	15/47 (31.9%)	-
55-64 years	27/84 (32.1%)	-
65-74 years	23/64 (35.9%)	30.6%
>74 years	3/8 (37.5%)	-
9-11mm		
All	15/227 (6.6%)	-
22-34 years	0/9 (0%)	-
35-44 years	0/15 (0%)	1.7%
45-54 years	2/47 (4.3%)	-
55-64 years	7/84 (8.3%)	-
65-74 years	5/64 (7.8%)	11.4%
>74 years	1/8 (12.5%)	-
≥12mm		
All	1/227 (0.4%)	-
22-34 years	0/9 (0%)	-
35-44 years	0/15 (0%)	1.2%
45-54 years	0/47 (0%)	-
55-64 years	1/84 (1.2%)	-
65-74 years	0/64 (0%)	5.4%
>74 years	0/8 (0%)	-
**Chronic periodontitis** (CPI >4)		
**All**	90/227 (39.6%)	
22-34 years	2/9 (22.2%)	
35-44 years	7/15 (46.7%)	
45-54 years	19/47 (40.4%)	
55-64 years	36/84 (42.9%)	
65-74 years	24/64 (37.9%)	
>74 years	2/8 (25.0%)	

Supplementary Table S4. Utilisation of dental healthcare services by rheumatoid arthritis patients and community subjects.

Dental checkup	Number (Percentage)	Oral Health Survey 2011
Regular visit		
Every 6 months	13/183 (7.1%)	27.5%
Every 1-2 years	71/183 (38.8%)	15.2%
>2 years	8/183 (4.4%)	13.6%
Visit only when dental symptoms arise	81/183 (44.3%)	43.7%
Never visit a dentist	10/183 (5.5%)	0%

Supplementary Figure S1. Proportion of rheumatoid arthritis patients having positive DMFT (A), decayed teeth (B), missing teeth (C) and filled teeth (D) by age groups (line representation), and by adult and elderly categories (bar representation), in comparison with the age-matched community subjects in Oral Health Survey 2011.

(A)

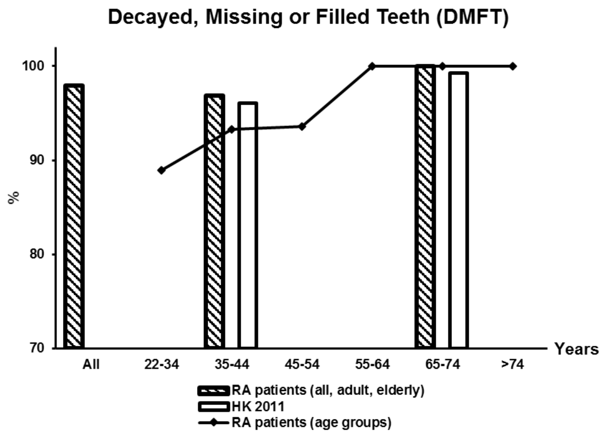

(B)

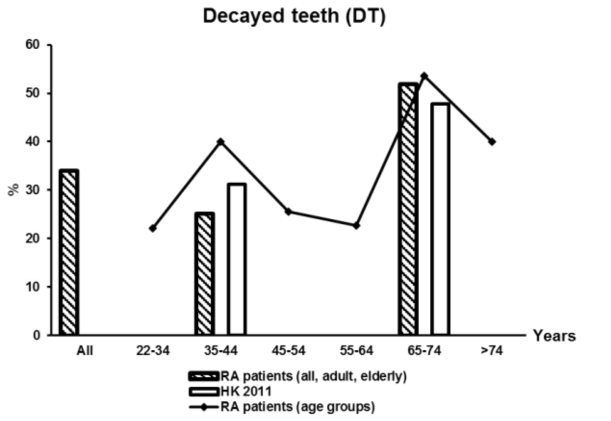

(C)

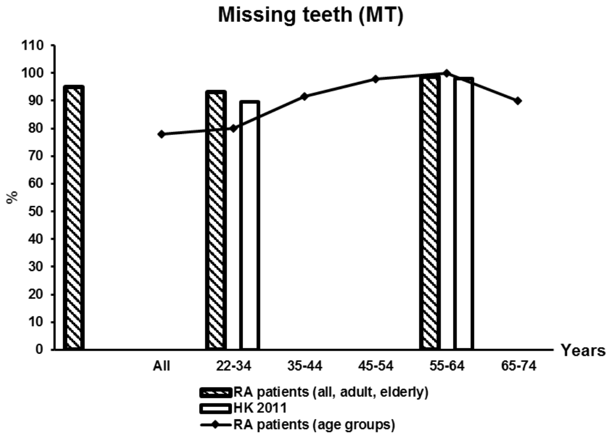

(D)

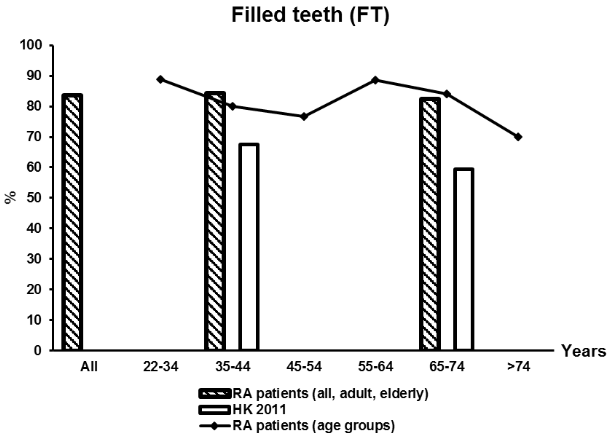

Supplementary Figure S2. Highest pocket depth (A) and maximum loss of attachment (B) in rheumatoid arthritis patients by 10-year age groups in comparison with the age-matched community subjects in Oral Health Survey 2011.

(A)

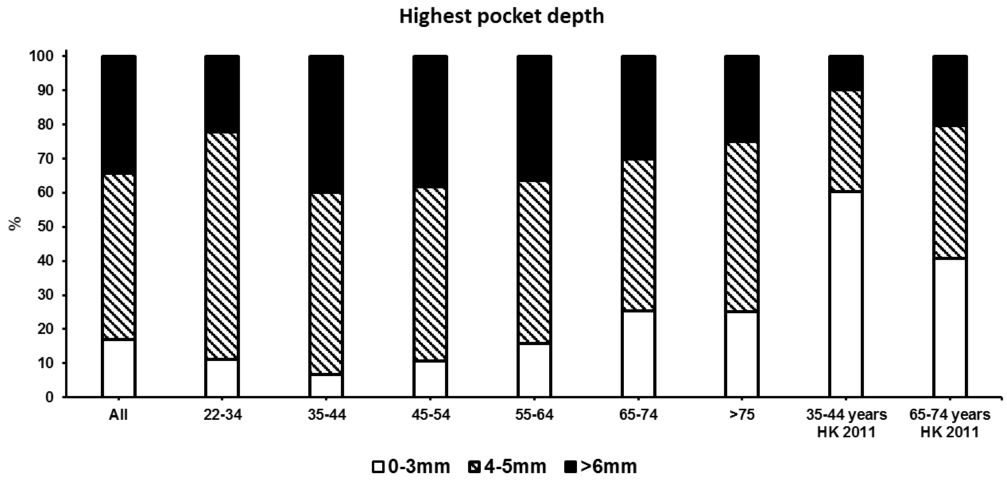

(B)

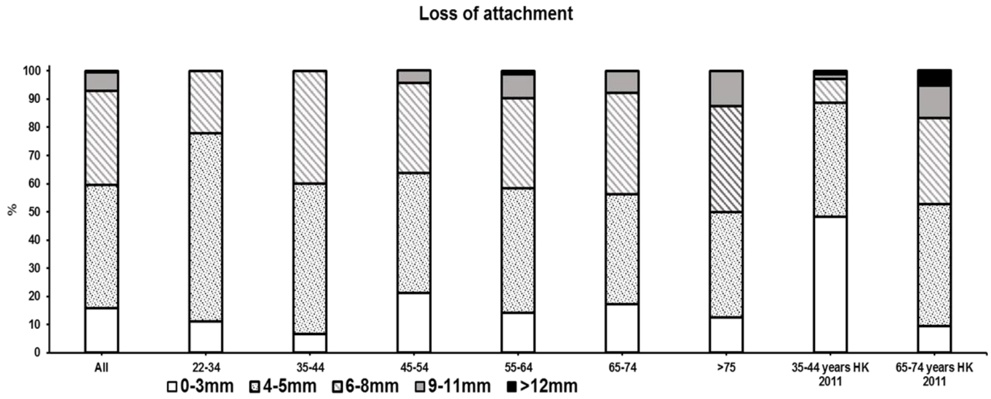

Supplement: Supplementary Materials — Supplementary Table S1: number of teeth in rheumatoid arthritis patients and age-matched community subjects. Supplementary Table S2A: dental outcomes of rheumatoid arthritis patients and age-matched community subjects by adult and elderly categories. Supplementary Table S2B: dental outcomes of rheumatoid arthritis patients and age-matched community subjects by 10-year age groups. Supplementary Table S3A: periodontal outcomes of rheumatoid arthritis patients and age-matched community subjects by adult and elderly categories. Supplementary Table S3B: periodontal outcomes of rheumatoid arthritis patients and age-matched community subjects by 10-year age groups. Supplementary Table S4: utilisation of dental healthcare services by rheumatoid arthritis and community subjects. Supplementary Figure S1: proportion of rheumatoid arthritis patients having positive DMFT (A), decayed teeth (B), missing teeth (C), and filled teeth (D) by age groups (line representation) and by adult and elderly categories (bar representation), in comparison with age-matched community subjects in the 2011 Oral Health Survey. Supplementary Figure S2: highest pocket depth (A) and maximum loss of attachment (B) in rheumatoid arthritis patients by 10-year age groups in comparison with age-matched community subjects in the 2011 Oral Health Survey. [file 8710880.f1.pdf]
